# Keyword index

**DOI:** 10.1054/bjoc.2001.1915

**Published:** 2001-06


					Aberrant crypt foci 90
N-Acetylcysteine 558
Acinar cancer 253
Acute lymphoblastic
leukaemia 11
Acute myeloid leukaemia 680
Adenocarcinomas 813, 969,
1199
Adenomas 1354
Adenovirus 1252
ADEPT 1671
Adhesive molecules 802
Adjuvant chemotherapy 186,
878
Adjuvant therapy 263, 1146
Administration routes 1564
-Adrenergic receptors 1648
Adults 1193
Advanced breast cancer 1172
Advanced colorectal cancer
651, 1023
Advanced Hodgkin s disease
1293
Adverse effects 697
AG2034 308
Allelotypes 321
Allelotyping 253
All-trans retinoic acid 1571
Alternating chemotherapy 1293
Alternative opioids 587
Alternative splicing event 1087
Alveolar type II pneumocytes
813
Amifostine 19, 313
Aminolevulinic acid 33
Amphiregulin 936
Amplification 824
Ampulla of Vater cancer 253
Amsterdam criteria 1084
ANA gene 754
Anaemia 17 (Suppl 1),
24 (Suppl 1), 31 (Suppl 1)
Analytical variations 1301
Androgen-independent
progression 859
Androgens 760
Androgens, and breast cancer
760
Angiogenesis 626, 832, 844,
1322, 1354
Animals 1681
Anthracyclines 550, 1437
Anthropomorphic factors 1466
Antiangiogenesis 1252
Antibodies 1671
Antibody-directed enzyme
prodrug therapy 1253
Anticancer drugs 100
Anticancer drug discovery 1424
Anti-idiotypic antibodies 1443
Anti-oestrogens 897, 1047
Anti-oestrogen resistance 686
Antioxidants 1397
Antisense oligonucleotides 768
Antivascular action 565
Antivascular effects 290
Anxiety 8
Anxillary lymph nodes 33
Aphidicolin 680
Apoptosis 87, 100, 263, 520,
670, 836, 851, 859, 951, 1099,
1126, 1135, 1265, 1272, 1330,
1412, 1551, 1571
Apoptotic index 651
Arrays 1512
Ascorbic acid 1544
Ashkenazi Jews 475
Aspirin 965
Astrocytoma 429
A49T 1344
Attitudes 413
A-type lamins 512
Atypical hyperplasia 209
Autocrine 926
Autologous stem cell
transplantations 1348
Autosomal recessives 429
Barrett's oesophagus 748
Basal cell skin carcinomas 512
Bax expression 651
BBR 3464 1387
bcl-2 263, 686, 1397
bcl-2 expression 651
bcl-2 homologues 920
Benign breast lesions 1064
Benign prostatic hyperplasia
1512
Betel quid chewing 709
BID 670
Bioavailability 42
Biochemical modulation 1023
Biochemotherapy 1036
Biological activity 3 (Suppl 1)
Biological variations 1301
Biphosphonates 1126
Birth weight 1193
Births 406
BIS20x3 1115
Bi-specific antibodies 1115
Bisphosphonates 951, 1047
Bladder 270, 1242
Bladder cancer 321, 558, 1330
Bleomycin 565, 1272
Blindness 397
bmi-1 1372
Body mass index 975
Bone marrow endothelium 1417
Bone metastases 1586
Bone mineral density 1047
Bone pain 297
Bone sialoprotein 344
BPH 1512
Brain cancer 990
Brain metastases 903
Brain tumours 1107
BRCA1 475, 704
BRCA2 193
BRCA2 475, 478, 704
Breast cancer 126, 157, 397,
435, 493, 700, 704, 760, 783,
802, 897, 915, 994, 1064,
1095, 1126, 1156, 1188, 1252,
1258, 1348, 1437, 1466, 1472,
1488, 1577, 1591, 1677
Breast cancer, advanced 1172
Breast cancer cells 936
Breast cancer prognosis 760
Breast cancer screening 423
Breast feeding 1472
Breast neoplasms 1047, 1308
Breast tumours 33
Breast-conserving therapy 539
Breast-ovarian cancer 482
Bryostatin 1 465
BTAK 824
N-Butyldeoxynojirimycin 1107
B-type lamins 512
Budding 1317
Bystander effects 674
CA 125 1301
Calpain 946
Cancer 8, 11 (Suppl 1), 17
(Suppl 1), 24 (Suppl 1), 31
(Suppl 1), 48, 126, 179, 406,
1193, 1207, 1219, 1463
Cancer cachexia 1135
Cancer cell invasion metastasis
1664
Cancer chemotherapy 550
Cancer gene therapy 1677
Cancer genetics 435
Cancer incidences 1215
Cancer modelling 388
Cancer risks 594, 1466
Cancer screening 360
Cancer in situ 791
Cancer vaccines 915
Cancers, human 193
Capecitabine 1437
Capillary counting 836
Carbohydrates 3 (Suppl 1)
Carbohydrate determinants 1258
Carboplatin 38, 170
Carcinomas 253, 270, 631,
1076, 1512, 1630
Carotene 728
Case-control studies 120, 134,
141, 697, 1472
Caspases 1135
Caspase 3 activity 851
Caspase 9 851
Castration 859
Catenins 1656
-Catenin 209
Caudal related homeobox genes
218
Causes of death 700
CD20 1115
CD44 244
CD44v6 244
CD55 (DAF) 80
cDNA array 352
CDX2 218
CDX2 1314
Cell cycles 1242, 1372, 1571
Cell cycle arrest 1640
Cell growth control elF3 1520
Cell transformation 374
Cell-cycle G0
/G1
arrest 768
c-erbB-2 1377
c-erbB-3 1377
c-erbB-4 1377
Cervical cancer 24 (Suppl 2),
360, 791, 892, 1616
Cervical dysplasia 796
Cervical intraepithelial neoplasia
796
Cervical smears 360
Cervix 1058, 1219
CGH 202, 499
Chemical cleavage of mismatch
482
Chemoprevention 90
Chemoprotection 19
Chemotherapy 11 (Suppl 1), 17
(Suppl 1), 19, 24 (Suppl 1), 31
(Suppl 1), 170, 303, 1150,
1179, 1391, 1447, 1577
Childhood cancer 141, 413,
1460
Childhood leukaemia 406, 1002
Childhood mortality 1460
Childhood non-Hodgkin's
lymphoma 1002
Children 604, 697, 1215
Chimeric PCR primers 482
ChlVPP/PABIOE 1293
Chromogranin A 636, 808
Chromosomal location 237
Chromosomal rearrangement
535
Chromosome 8 72
Chromosome 19p13 493
Chromosome 21 754
Chromosome aberrations 776,
892
Chromosome instability 489
Chronic disease 24 (Suppl 1)
Chronic hypoxia 1122
CIN 360
Circulating tumour cells 631
Cisplatin 565, 1387
1695
British Journal of Cancer (2001) 84(12), 1695-1698
(c) 2001 Cancer Research Campaign
doi: 10.1054/ bjoc.2001.1915, available online at http://www.idealibrary.com on
Keyword index to Volume 84
Key to abbreviations:
(Suppl 1) Supplement 1; (Suppl 2) Supplement 2
Clear cell sarcomas 535
Clinical decision-making 1172
Clinical Practice Guidelines 1
(Suppl 2), 4 (Suppl 2), 6
(Suppl 2)
Clinical samples 768
Clinical studies 11 (Suppl 1)
Clinical trials 1424
Clonogenic survival 100
c-Met 864
c-myc 72
c-myc 813
Codon 72 polymorphism 739
Cognitive dissonance reduction
1577
Cohorts 113
Coincidences 1084
Collagenases 1076
Colon cancer 66 (Suppl 2), 90,
844, 1412, 1535
Colon cancer cell lines 579
Colon neoplasia 1265
Colorectal adenocarcinoma
1354
Colorectal cancer 94, 196, 218,
232, 417, 475, 520, 600, 611,
892, 910, 969, 1314, 1317,
1443, 1497, 1671
Colorectal cancer, advanced
651, 1023
Colorectal neoplasia 722, 1308,
1477
Combination immunotoxin
therapy 571
Combined therapy 460
Combretastatin 832
Comet assay 1671
Communications 1011
Comparative genomic
hybridization 202, 499
Conservative surgery 164
Continuous intravenous infusion
1043
Contrast media 1681
c-Src tyrosine kinase 196
CTL 94
Cutaneous melanoma 81
(Suppl 2)
Cyclic AMP 1648
Cyclin B 283
Cyclin D1
270, 1064
Cyclin E 1242
Cyclin-dependent kinases 283,
1391
Cyclin-dependent kinase 1 283
Cyclo-oxygenease-2 335
Cyclophosphamide 1591
Cyst fluid 1363
Cytarabine 157
Cytochrome c 1099
Cytokines 659, 1122
Cytoprotection 313
Cytosine arabinoside 680
Cytotoxic T lymphocytes 1052,
1564
Cytotoxicity 106, 1535
Darbepoetin- 3 (Suppl 1),
11 (Suppl 1), 17 (Suppl 1),
24 (Suppl 1), 31 (Suppl 1)
De novo carcinoma 1497
Deglyco-bleomycin-A2
1272
185delAG 475
Deletions 193
617delT 475
Dendritic cells 1564
5-Deoxy-5-fluorouridine 1677
Deoxyuridine 11
DESC1 237
Detection mode 423
Detection of psychiatric
morbidity 1011
Dexrazoxane 959
Diagnosis 406, 1512
Diethylstilbestrol 126
Diets 728
Differential expression 237
Differentiation 520, 1528
Differentiation inducers 1556
DNA adducts 226
DNA damage 106
DNA methylation 754
DNA repair 489, 680, 1387
DNA vaccine 374
Docetaxel 170, 470, 1571
Dose-repsonse relationship
1544
Doxorubicin 367, 959, 1591
DPC4/Smad4 253
Drugs 1544
Drug combination 579
Drug delivery 157
Drug resistance 186, 680, 959,
1391
Drug uptake 1528
Ductal carcinoma in situ 539
EB1089 686
E-cadherin 1656
EDTA 452
E6/E7 oncogenes 374
EGCG 844
Electrochemotherapy 565, 1272
Electromagnetic fields 697
Electroporation 565, 1272
Embryonal carcinoma 340
Endocrine tumours 253
Endometrial cancer 31 (Suppl
2), 126, 209, 545, 969, 975
Endometrium 897
Endothelial cells 565
Endothelial growth factor
receptors 926
Endothelium 80
England 1215
Epidemiology 122, 392, 400,
413, 910, 994, 1193, 1482
Epidermal growth factor 936
Epidermal growth factor
receptors 1322, 1377
Epidermoid cancers 37
(Suppl 2)
Epidoxorubicin 878
Epigallocatechin gallate 844
Epithelial tumours of the thymus
51 (Suppl 2)
Epitope peptides 94
Epstein-Barr virus 783, 920,
1227
Epstein-Barr virus nuclear
antigens 920
ER 686
Erk-1 844
Erk-2 844
Erythropoietin 3 (Suppl 1), 24
(Suppl 1)
Erythropoietin mRNA 836
Erythropoietin receptor mRNA
836
Ethics 413
Etoposides 1453
European guidelines 587
European Organization for
Research and Treatment of
Cancer QLQ-H&N35 149
EWS-Fli1 768
Exercises 994
Expression 504
Extensive surgery 1602
Familial cancer 388, 429
Familial risks 969
Fas 1330
Fas-ligands 1330
Females 1188
Fenretinide resistance 1528
Fibroblast growth factors 1656
Fibroblast growth factor
receptors 1656
Fish consumption 1199
Flavonoids 1391
Flow cytometry 367, 626
Fluorescence in situ
hybridization 72
Fluorescence-assisted mismatch
analysis 482
Fluorescent single-strand
conformation polymorphism
1505
Fluorouracil 579
5-Fluorouracil 263, 611, 878,
1023, 1437, 1677
5-Fluorouracil pharmacokinetics
600
Folate analogue 308
Formalin fixed 1095
-Fucosyltransferase 1556
Fusion transcripts 1087
Gangliosides 1107
GARFT inhibitors 308
Gastric cancer 64, 186, 335,
400, 470, 824, 878, 965, 1602
Gelatinase 1076
Gemcitabine 1179
Gene expression 643
Gene therapy 1252
Genetic counselling 594
Genetic epidemiology 388
Genetic predisposition 776
Genotyping 783
Geographical variability 1482
GF120918 42
Glioblastoma 529
Glioma 429, 1107
Global indicators 1156
Glomerular filtration 452
Glucosyltransferase 1107
-Glucuronidase 550
Glucuronide 550
Glycogen phosphorylase 1497
Glycoprotein hormones 808
Glycosphingolipids 1107
Goitre 832
Growth inhibition 1640
Guidelines 796
Head and neck 149, 237, 504,
776
Health-related quality of life
149
Heat 120
Helicobacter pylori 965
Hepatitis B 1207
Hepatitis B virus 709
Hepatitis C virus 709
Hepatocarcinoma cells, human
1556
Hepatocellular carcinomas 74
(Suppl 2), 709, 881, 886, 982,
1377, 1640
Hepatocyte growth factor 864
Hepatocyte growth factor
antagonists 864
Hepatomas 743
HER2/neu 94
Hereditary breast cancer 116
Hereditary non-polyposis
colorectal cancer 1084
Heredity 388, 429
Hexadecylphosphocholine 1405
High-dose chemotherapy 313,
1348
Histological type 400
Histology 539
HLA-A24 915, 1052
HMG-CoA reductase inhibitors
886
HNPCC 1084
Hodgkin's disease 55 (Suppl 2),
381, 1227, 1339
Hodgkin's disease, advanced
1293
Hormones, female 722
Hormones and cancer 760
HPA 819
H-ras 374
hSNF5 199
hTERT 621
Human papillomaviruses 791,
796, 1616
Human papillomavirus 16 1058
Humans 1599
Hyperinsulinaemia 417
Hyperplasia 832
Hyperplasia, atypical 209
Hypoxia 626, 1070, 1280
Id3 352
Immune escapes 1330
Immunization 1207
Immunohistochemistry 270,
321, 539, 1095, 1377
Immunosuppression 1624
Immunotherapy 915, 1258,
1443
In situ hybridization 783
In vitro assay 558
In vitro-to-in vivo correlations
1424
In vivo proton magnetic
resonance spectroscopy 1016
Incidences 1084, 1207, 1463
Indirubin 283
1696 Keyword index
British Journal of Cancer (2001) 84(12), 1695-1698 (c) 2001 Cancer Research Campaign
Indirubin-3-monoxime 283
Infections 406, 1002
Information needs 48
Inherited predisposition to
cancer 475, 478, 892
Inhibitors 1535
INI1 199
5382insC 475
Interferons 611, 1036, 1146
Interleukin-1 329
Interleukin-2 1036
Interstrand crosslinks 1671
Intraductal papillary-mucinous
tumours 253
Intratumoral injections 443
Intraventricular therapy 1453
Invasiveness 276, 558, 802
Ionizing radiation 674, 776
Irinotecan 579
Isoprenyl transferases 1535
Isoprenylation 1535
Isothiocyanates 670
Italy 196
Jewis founder mutations 478
JNK 670
Juvenile polyposis 1314
K562 959
Kallikreins, human 643
Kallikrein gene 5 643
Ki-57 1064
Ki-67 340, 1242
K-ras 253, 1497
Kupffer cells 1265
LABC 1016
Lactation 1472
Laminin-5 659
Laser capture microdissection
783
Latency 920
Latent membrane protein 920
Latent membrane protein-1
1227
Lectins 819
Leucovorin 878
Leukaemia 697, 990
Leukaemia, acute lymphoblastic
11
Leukaemia, acute myeloid 680
Lewis antigen 1556
Li-Fraumeni syndrome 116
Liability 388
Lifestyle factors 417
Lipid mobilizing factor 1648
Lipoic acid 1544
Liposomal cisplatin 1029
Liver cirrhosis 1265
Local recurrences 244, 539
Locally advanced breast cancer
1016
Long-term survival 700
Loss of heterozygosity 218, 226,
352, 381, 493, 739, 754, 1630
Low risk breast cancer 164
Low-dose computed tomography
25
Lung cancer 25, 134, 392, 728,
892, 910, 1520
Lung neoplasia 1199
Lymph nodes 340
Lymphocytes 776, 1339
Lymphomas 990
MAC 15A murine tumours 290
Macrophages 626
Magnetic resonance imaging
691, 1681
Male breast cancer 482
Malignant cells 87, 836
Malignant melanoma of soft
parts 535
Malignant mesothelioma 49
(Suppl 2), 52
Manganese 1681
Manganese superoxide
dismutase 529
MAPK AP-1 926
Mass screening 1477
Matrilysin 1317
Matrix metalloproteinases 1076,
1664
Matrix metalloproteinase-1 276
Matrix metalloproteinase-2
1363
Matrix metalloproteinase-9
1363, 1488
MCF-7 686
MCF-7 human breast carcinoma
cell line 367
MDR1 1405
Medical lymphadenectomy 443
Medullary thyroid carcinoma
808
Megestrol 881
Melanoma 81 (Suppl 2), 535,
819, 1036, 1146, 1466
Melatonin 397
Membrane potential 1099
Meningioma 199
Menstrual cycles 714
Mesothelioma 49 (Suppl 2), 52
Meta-analysis 303, 611, 722,
1150, 1188
[131
I]-Metaiodobenzylguanidine
460
Metalloporphyrins 1681
Metastasis 819, 1036, 1265,
1280, 1317, 1417, 1610
Metastasis-related phenotypes
1556
Metastatic medulloblastoma
1453
Methotrexate resistance 11
Micronucleus 674
Microsatellites 253
Microsatellite instability 232
Microvessel density 1610
Migrants 1215
Migration 1564
Miltefosine 1405
Mitochondria 1099
Mitochondrial permeability
transition 1397
Mitomycin 1150
Mitomycin-C 1023
Mitotic cell deaths 1272
Mitoxantrone 859
MLH1 321
Monitoring 1301
Morphine 587
Mortality 392, 423
Mouse 1107
mRNA 276
MSH2 321
Mucin 1258
Multidrug resistance 959, 1405
Multiple myeloma 344, 621
Multiple primary cancers 435
Muscle proteolysis 1135
Muscle wasting 946
Mutations 199, 209, 335, 493,
704, 1344
Myelotoxicity 19
Myometrial invasion 545
NACT 1016
National surveys 1447
Natural killer/T-cell lymphomas
920
Neoadjuvant chemotherapy
1016
Neoplasia 1242, 1681
Neoplastic bone involvement
344
Neoplastic meningitis 157
Nested case-control studies 122,
975
Neuroblastoma 460
Neuroendocrine tumours 636,
808
Neuron-specific enolase 903
NIH 3T3 691
Nitric oxide 1122
Nitric oxide synthase 1122
NK4 864
Non-colon cancer 478
Non-Hodgkins lymphoma 113,
303, 465, 499, 1115
Non-small cell lung cancer 226,
903, 1150, 1179, 1372
Non-smokers 134
Non-steroidal anti-inflammatory
drugs 965, 1188, 1412

Np73L 1235
Nuclear lamins 512
Oesophageal adenocarcinoma
748
Oesophageal carcinoma 61
(Suppl 2), 276
Oesophageal neoplasia 120
Oesophagitis 120
Oestradiol 975
Oestrogens 126, 975
Oestrogen receptors 1064
Oestrogen receptor- 545
Oestrogen receptor- 545, 1095
OK-432 443
Oral administration 42
Oral cancer 739, 754, 1437
Oral contraceptives 722
Organ cultures 1076
Oropharyngeal cancers 37
(Suppl 2)
Osteopontin 951
Osteoprotegerin 951
Osteosarcomas 78 (Suppl 2),
951
Ovarian cancer 18 (Suppl 2),
126, 170, 352, 594, 643, 704,
714, 1043, 1301, 1363, 1387,
1624
Ovarian cancer cells 1551
Ovarian neoplasia 1308
Ovarian tumours 1528
Overall survival 1610
Overexpression 824
Oxaliplatin 579
P13K 1630
p14ARF 1372
p16 253, 504, 1372
p16 methylation 982
p21CIP1/WAF1
expression 1635
P27Kip1
1242
p38 MAPK 851
p53 253, 263, 529, 1052, 1070,
1497, 1505, 1551
p53 antibodies 57
p53 autoantibodies 52
p53 expression 651
p53 gene 739
p53 genes 335
p53 homologues 1235
p53 mutations 226, 1635
p73 antibodies 57
P73L 1235
p170 1520
Paclitaxel 38, 42, 604, 1126,
1166, 1179, 1591
Paediatrics 1029
Palliative chemotherapy 1172
Pamidronate 1586
Pancreas 253
Pancreatic adenocarcinoma 1656
Pancreatic cancer 864
Papillomavirus 1058
Papillomavirus, human 374, 1219
Paraffin-embedded materials
1095
Parity 1219
Participation 413
Paternal occupational contacts
1002
Patient information 8
Patients' distress, oncologists'
estimations of 179
Patients' preferences 48
PBSC transplantation 313
PCR 1505
Peptides 1052
Perinatal factors 1466
Peroxisome proliferator-
activated receptor- 1640
Peutz-Jeghers syndrome 1314
P-glycoprotein 42, 367, 959
Phaeochromocytomas 636, 808
Pharmacoeconomics 313
Pharmacokinetics 3 (Suppl 1),
11, 11 (Suppl 1), 1029, 1453
Phase I 604, 1029
Phosphocholine 691
Photodynamic diagnosis 33
Photodynamic therapies 33, 1099
Photosensitizers 33
Phthalocyanine Pc 4 1099
Picibanil 443
PIN 1512
Keyword index 1697
British Journal of Cancer (2001) 84(12), 1695-1698
(c) 2001 Cancer Research Campaign
PK11195 1397
Plasma cells 621
Ploidy 232
Ploidy patterns 824
Pluronic F68 90
pO2
histographs 626
Point mutations 760
Polarographic oxygen electrodes
and sensors 1070, 1280
Poly(ADP-ribose) polymerase
106
Polycomb group genes 1372
Polyethylene glycol 90
Polymerase chain reaction 783
Polymorphisms 743, 760
Population pharmacokinetics
452
Post-treatment cervical
intraepithelial neoplasia 796
Postmenopausal osteoporosis
1047
Pravastatin 886
Pre-cancerous lesions 1354
Pre-operative immunotherapy
443
Predictive assay 1070
Preferences 1577
Prenatal environment 1193
Preoperative chemotherapy 1016
Prevention 994
Primary fibroblasts 674
Probability models 704
Prognosis 64, 186, 244, 276,
344, 423, 819, 903
Prognostic factors 1488, 1602
Prognostic markers 643
Progression criteria 1301
Proliferation inducers 1556
Prostate cancer 202, 297, 859,
1344, 1076, 1417, 1571
Prostate epithelia 1417
Prostate specific antigens 760
Prostatic intraepithelial neoplasia
202
Proteases 936
Protein breakdown 946
Protein kinase C inhibitors 465
Protein synthesis 1648
Proteolysis-inducing factor 1599
Proto-oncogenes 196
Protracted infusion
5-fluorouracil 600
[31
P]-spectroscopy 691
Psychiatric morbidity, detection
of 1011
Psychological co-morbidity 179
Psychological distress 594
Psychotherapeutic support 179
PTEN 1630
PTEN 748
10q23 748
12q12-14 499
Quality of life 297, 1156
Quantitative RT-PCR 1664
QLQ-H&N35 149
Radiation therapy 489, 851
Radionuclide imaging 367
Radioresistance 489
Radiosensitivity 892
Radiosensitization 1122
Radiotherapy 8, 31 (Suppl 1),
164
Radon 134
Railroads 697
Randomized controlled trials 8,
878, 1156
RANKL 951
Ras 691
ras mutations 982
Ras superfamily 1535
Reactive oxygen species 529,
1397
Real-time quantitative
polymerase chain reaction 783
Rearrangements 193
Recombinant human
erythropoietin 31 (Suppl 1)
Rectal cancer 69 (Suppl 2)
Recurrences 1602
5--Reductase 760, 1344
Registration 1207
Registries 435
Renal function 452
Reproductive factors 714
Response to treatments 651,
1156
Retinoblastoma endocrine
tumours 990
Retinoblastoma protein 520
Retinoic acid receptors- 1528
Retinoids 1528
Retinol 728
Reverse transcriptase
polymerase chain reaction 535
Reviews 3 (Suppl 1)
Right colon 969
Risk factors 709
Risks 417, 1199
Rituximab 1115
RIZ(PRDM2) 743
Rodent tumours 1280
RT-PCR 72, 1087
Rural population mixing 910,
1002
SAFB 493
Salivary gland tumours 42
(Suppl 2)
Sarcomas 535, 1635
SART3 antigens 915
Scale comparisons 1156
Schedule of administration 1023
Scotland 1002
Scottish trials 1146
Screening 25, 1616
Seasons 406
Segregation analyses 429
Selectivity 87
Sensitivity 1477
Sequence variations 791
Sequential chemotherapy 470
Serine protease 237
Severe combined
immunodeficient mouse 571
Sex hormone-binding globulin
975
SF-36 149
Shared decision-making 1577
Short-term infusion 604
Small cell lung cancer 19, 38,
1166, 1447
SMARCB1 199
Smoking 141, 226, 1219
Social class 179
Social support 179
Sodium phenylacetate 802
Soft-tissue sarcoma 244, 1070,
1610
Soft-tissue tumours 535
Sojourn times 1477
Solid pseudopapillary tumours
253
Solid tumours 17 (Suppl 1)
SOR Clinical Practice Guideline
Program 1 (Suppl 2), 4 (Suppl
2)
SOR project methodology 8
(Suppl 8)
South Asians 1215
Southern blot 193
SPI-77 1029
Squamous cell carcinoma 237,
504, 1235
Squamous cell carcinoma
antigens 851
Squamous intraepithelial lesions
1058
SRD5A2 760
Staging 910
Statistical models 1477
Stimulation 1339
Stroma 80
Stromal fibroblasts 1664
Stromelysin-1 659
Strontium-89 297
-Subunit of glycoprotein
hormones 808
Suramin 558
Survival 19, 232, 270, 303, 886,
1070, 1602
Survival analysis 643
Survivors 149
Sweden 113
Synovial sarcomas 1087
Systematic reviews 1150
Tamoxifen 881, 897
Taxoids 1437
T-cell leukaemia 571
TCho 1016
99m
Tc-sestamibi 367
TcR/CD3-zeta 1624
Telomerase 504, 621, 631, 1551
Telomere length 1348
Temporal trends 1482
Testicular cancer 1482
ThinPrep cytology 360
Thioctic acid 1544
Thymal tumours 51 (Suppl 2)
Thymidylate synthase 186
Thyroid cancer 1586
Time trends 400
TNM classification 64
Tobacco 392
Topoisomerase I 106
Topoisomerase II 106
Topotecan 1043, 1166
Toremifene 897, 1047
Total choline 1016
Toxicity 313
TP53 mutations 116
Transfection 768
Transformation 499
Transfusions 17 (Suppl 1)
Transitional mucosa 1497
Translation initiation 1520
Trypsin 1363
Tuberculosis 113
Tumoral immune response 57
Tumours 832
Tumour antigens 57
Tumour burden 344
Tumour cells, cultured 1544
Tumour expression 80, 1599
Tumour invasion 1317
Tumour oxygenation 1070
Tumour progression 202
Tumour recurrence 1610
Tumour suppressor genes 352,
381, 493
Tumour vascular architecture
1354
Tumour virology 122
Tumour-associated trypsin
inhibitors 1363
Tumour cell vaccine 374
Twins 1460, 1463
Tyrosine phosphorylation 670
UFT 263
Urban 910
Uridine phosphorylase 1677
Urinary bladder cancer 329,
1505
Urokinase 329
Vaccine efficiency 1564
Variant liver oestrogen receptor
transcripts 881
Variations 1058
Vascular endothelial growth
factor 80, 844, 1252, 1322,
1610
Videotapes 8
Vinflunine 290
Vinorelbine 290, 1437
Vinyl chloride 982
Viral interleukin-10 920
Visual impairment 397
Vitamin C 1544
Vitamin D analogue 686
Vitamin E analogue 87
Vitamin K 1544
Volume of clinical activity 1308
Weekly chemotherapy 1166
Weight loss 1599
Wilms' tumour 990
Women 392, 1193
Zeta chain 1339
1698 Keyword index
British Journal of Cancer (2001) 84(12), 1695-1698 (c) 2001 Cancer Research Campaign

				

